# Tuning in: how interoception shapes body image across early adolescence

**DOI:** 10.1098/rstb.2025.0159

**Published:** 2026-11-10

**Authors:** Mariana Von Mohr, Rosie Donaghy, Vivien Ainley, Imogen Moore, Abigail Agyemang, Manos Tsakiris

**Affiliations:** ^1^ Department of Psychology, Royal Holloway University of London, London, UK; ^2^ Centre for the Politics of Feelings, School of Advanced Study, University of London, London, UK; ^3^ School of Psychology, University of Kent, Canterbury, UK; ^4^ School of Psychology, University of Surrey, Guildford, UK; ^5^ Department of Psychology, University of Cambridge, Cambridge, UK

**Keywords:** interoception, body image, adolescence, puberty, interoceptive accuracy

## Abstract

Early adolescence is a critical time marked by dynamic changes in physiology, appearance and social environment. These changes are also reflected in how adolescents perceive themselves, including their bodies. The transition through pubertal stages allows us to consider the development of the self from the perspective of how body perception, both from within and from the outside, changes. Although research in adults and post-puberty adolescents suggests that lower interoceptive accuracy is associated with greater body-image concerns, how these factors interact during puberty remains unknown. We tested a sample of 181 girls (ages 10–14 years) spanning across a broad range of pubertal stages, on measures of interoceptive accuracy and behavioural and self-report measures of body image. Lower interoceptive accuracy predicted greater body-image dissatisfaction. Moreover, this relationship was moderated by pubertal stage, showing that the protective effect of higher interoceptive accuracy against body-image dissatisfaction was stronger during more advanced stages of puberty. Our findings show that interoceptive influences extend from the basic multisensory integration to conscious attitudes about our body during a critical developmental period, highlighting the positive role that better interoception can play in mental health.

This article is part of the discussion meeting issue ‘From being to identity: analysis and synthesis of the self’.

## 1. Introduction

Our sense of self begins with bodily awareness, which arises from the integration of signals coming both from within the body and from its external appearance [1]. We perceive our bodies externally through exteroceptive senses such as vision and touch, which shape how we see ourselves in the world. This external perspective gives rise to *body image*, broadly defined as the mental representation of our body's appearance and related attitudes, perceptions, affects and thoughts [2]. Simultaneously, we are continuously informed by internal bodily signals, such as heartbeats, respiration and visceral sensations. This internal dimension, known as interoception, refers to the sensing of the body's physiological state [3]. Interoception plays a key role in shaping how we feel, regulate emotions and maintain a coherent sense of self [4]. It is recognized as a multidimensional construct with two conscious components: interoceptive accuracy (i.e. the objective ability to detect internal signals) and interoceptive sensibility (i.e. the subjective tendency to attend to those signals) [5]. Although traditionally studied in isolation, interoceptive and exteroceptive signals are increasingly understood as interactive, contributing together to a rich, multisensory representation of the bodily self [1,3,6]. The interplay between these two aspects of body-awareness, namely body image and interoception, and their impact on well-being is therefore crucial and has begun to be studied across the lifespan (e.g. [7–14]). However, little is known about how this interplay varies during sensitive developmental periods such as puberty, a stage marked by rapid physical, neural, hormonal and social changes [15]. Focusing on this relationship across pubertal stages can help us understand how a healthier body image is constructed, maintained or altered before body-image concerns may even begin to be fostered.

Building on this integrative perspective, studies on adults show that reduced interoceptive abilities are linked to a more negative perception of one's body, including body dissatisfaction, disturbances in body image and self-objectification (e.g. [7,12,13]). Related theoretical accounts [16] suggest that reduced interoceptive accuracy (i.e. one's diminished ability to perceive internal bodily signals) leads to a greater reliance on exteroceptive cues, such as visual appearance, to form body representations. According to the competition of cues hypothesis [17,18], focusing on internal stimuli can decrease the attention available for external stimuli, and vice versa [19]. This imbalance causes individuals to prioritize external information over internal bodily states when evaluating their bodies, making them more susceptible to body-image dissatisfaction. Within a predictive coding framework, this imbalance reflects the brain's reduced precision-weighting of interoceptive signals, allowing exteroceptive input to dominate perceptual inference. As a result, poor interoception contributes to a sense of bodily self that is dominated by exteroceptive, particularly visually driven representations [20,21]. Although interoception engages the self as the subject of the interoceptive experience (e.g. I feel this way), the exteroceptive facet of body-awareness often emphasizes an objectification of the self (e.g. this is how I look). This greater dependency on exteroceptive signals, particularly visual ones, may lead to an increased emphasis on the body's visual appearance. This in turn, can lead to its objectification [7] and increased vulnerability to societal ideals of beauty, resulting in idealized or distorted views of one's own body. Indeed, a tendency to rely on exteroceptive signals has been linked to disturbances in body image and to eating disorders [22–24]. Relatedly, altered activity in the insula, which is a key interoceptive hub, has been observed in individuals with body-image disturbances, consistent with disrupted internal bodily processing [25]. Conversely, individuals with greater interoceptive ability may be more apt to maintain a positive body image because they prioritize internal (physiological) signals (e.g. how I feel) as opposed to how the body is externally perceived (e.g. how I look).

Despite this growing body of adult research, our understanding of the relationship between interoception and body image during sensitive developmental periods, such as puberty, is limited. To the best of our knowledge, only three studies have examined the association between interoception and body image in adolescents, finding that higher interoceptive accuracy is related to less body dissatisfaction [9] and higher interoceptive sensibility is positively associated with positive body image [10] and negatively to body dissatisfaction [12], with some facets of the latter being mediated by intolerance of uncertainty. Additionally, one recent study in adolescents reported that the relationship between body image and mental health is mediated by interoceptive sensibility and body functionality (i.e. thoughts, feelings and perceptions about what one's body can do), highlighting the importance of bodily awareness and functionality in supporting psychological well-being [13]. However, these studies focused on adolescents ages between 13 and 16 [10], 14 and 15 [13], and 16–19 [9,12] years old, probably capturing individuals who had already undergone significant pubertal changes. As such, if and how interoception influences body image in earlier sensitive periods, such as early adolescence, and how this relationship unfolds across this developmental window, remains unknown.

The emergence of adolescence occurs around the onset of puberty [15], a critical period of sweeping hormonal, physical, psychological and social changes [2,26]. Developmental neuroscience shows that adolescence is marked by dynamic alterations in brain structure and function, with important implications for self-related processes [15]. Notably, sub-regions of the anterior insula (a central hub of the interoceptive network) follow distinct maturation trajectories across early to mid-adolescence [27], suggesting a neural basis for evolving bodily awareness. As self-processing circuits mature, early adolescence is marked by heightened self-consciousness and greater susceptibility to peer influence [15,28]. Given concurrent physical transformations occurring during puberty (e.g. in females: development of breasts and hair growth, onset of menses), this heightened self-focus may render adolescents particularly vulnerable to body-image concerns. Moreover, because bodily appearance is rapidly and unpredictably changing during puberty, and the adolescent self becomes more socialized and subject to social influences and peer pressure, internal bodily awareness—that is, interoception—may be even more important for maintaining a stable sense of body and self. Supporting this view, evidence identifies the pubertal transition as the period of steepest acceleration in body dissatisfaction and disordered-eating symptoms, particularly among girls [29–31]. Therefore, puberty could be a particularly vulnerable period where individuals may develop body-image concerns. Whether or not they do so may also depend on the awareness of their body from within, in other words, on their interoception. Consistent with this literature, the present study focused on girls, as body dissatisfaction during adolescence has been extensively documented in female samples, and pubertal bodily changes may be particularly relevant to body-image processes in girls [2,26,29–31].

Thus, in this cross-sectional study, we investigated the relationship between interoceptive accuracy and negative body image, namely body dissatisfaction, in 10–14-year-old girls across different stages of puberty, spanning early to more advanced pubertal stages. We used a body-image assessment software (BIAS) [32] and a body-image questionnaire (BIQ) [33] to examine body-image dissatisfaction at two related but distinct levels, namely a body-representation/adjustment level, given that this is a behavioural task that allows us to quantify body-image distortions and body dissatisfaction, and an explicit self-report level, respectively. We hypothesized that higher interoceptive accuracy would be associated with lower body dissatisfaction, and that this association would be moderated by pubertal stage, such that the protective effects of interoceptive accuracy would be stronger at more advanced stages of puberty. These hypotheses were tested in parallel across both outcome measures, allowing us to examine whether this pattern holds at both a body-representation level and an explicit self-report level. As discussed above, theoretical accounts suggest that individuals who tend to rely more on exteroceptive than interoceptive cues may be more prone to body image disturbances [7,16,19,21]. Because puberty involves many physical and physiological changes, to maintain a stable and healthier body image, individuals may need to rely more on good interoceptive awareness to help them navigate these changes across this sensitive period. If this is true, individuals with higher interoceptive accuracy should show less body dissatisfaction at more advanced pubertal stages, whereas those with lower interoceptive accuracy would show greater body dissatisfaction.

## 2. Material and methods

### (a) Participants

A total of 181 girls (*M* age = 11.89, s.d. = 1.25; range = 10–14 years) were recruited from primary and secondary schools in southwest England. Ethical approval was obtained from the Ethics Committee of Royal Holloway, University of London.

### (b) Design

This was a cross-sectional study examining whether interoceptive accuracy and pubertal stage predict body dissatisfaction, and whether pubertal stage moderates the association between interoceptive accuracy and body dissatisfaction. Because the design was cross-sectional, these analyses addressed between-subject differences across pubertal stages rather than within-person developmental change. Interoceptive accuracy and pubertal stage were included as continuous predictors (mean-centred), with their interaction term testing moderation. Body dissatisfaction was operationalized using two related but distinct outcome measures: (i) the BIAS, indexing discrepancy between perceived and desired body representations at a behavioural/adjustment level, and (ii) the BIQ, indexing explicit self-reported self-ideal body discrepancy.

Body mass index (BMI) and heart rate were included only as secondary covariates in sensitivity analyses and were not treated as primary theoretical predictors or moderators. Additional robustness analyses included age as a covariate owing to its correlation with pubertal stage.

### (c) Procedure

Written consent was obtained from both parents/guardians and participants. Testing took place individually in a school classroom. After explaining the procedure, participants completed the heartbeat counting task (HCT) [34], the body-image dissatisfaction task using the BIAS [32], and the BIQ [33], followed by the pubertal developmental scale (PDS; girls version) [35].

## 3. Material and measures

### (a) Body-image dissatisfaction

Behavioural body dissatisfaction was assessed using the BIAS [32], a tool with demonstrated validity and reliability [36]. Participants independently distorted different body parts of a female figure on the screen to create a whole-body representation of how they think they look (*perceived body*) and, separately, how they would like to look (*desired body*). Task order was counterbalanced.

The BIAS calculates average values across body parts for perceived and desired bodies. Dissatisfaction scores reflect the absolute difference between these values. Standard values are anchored at 100; values below 100 indicate thinner selections, greater than 100 indicate heavier ones. For example, a perceived score of 95 and a desired score of 93 yield a dissatisfaction score of 2. Higher scores indicate greater body dissatisfaction. Data from 12 participants were missing owing to technical issues. The mean dissatisfaction score was 3.81 (s.d. = 4.23).

### (b) Body-image questionnaire

The BIQ [33] is an 11-item self-report measure of self-ideal body discrepancy across traits like weight, muscle tone and facial features. For each item, participants rate how closely they match their ideal (0 = exactly as I am; 3 = very unlike me) and how important the trait is (0 = not important; 3 = very important). A composite score is calculated by weighting each discrepancy by its importance, ranging from –3 (full congruence on important traits) to +9 (maximum discrepancy on valued traits). Higher scores indicate greater self-ideal disparity. The BIQ has good reliability [33]; in our sample, Cronbach's alpha = 0.83. BIQ data from four girls in our sample were missing owing to technical errors. On average, the BIQ score was 0.99 (s.d. = 1.38).

### (c) Interoceptive accuracy

We assessed interoceptive accuracy using the HCT [34]. Without taking their pulse, participants silently counted felt, not guessed [37], heartbeats across six pseudorandomized intervals (25, 30, 35, 40, 45 and 50 s), signalled by ‘start’ and ‘stop’ audio cues. Simultaneously, actual heartbeats were recorded using a finger pulse oximeter (Xpod 3012LP, NONIN) connected to a laptop running *I*nteroceptive*T* esting*2.2* (August 2015, Dept. Psychiatry, Brighton & Sussex Medical School, developed by c.gould@bsms.ac.uk).

In each trial, reported and actual heartbeats were compared to calculate an index of interoceptive accuracy, using the following equation: 1 − (| *n*beats_real_ − *n*beats_reported_ |)/((*n*beats_real_ + *n*beats_reported_)/2). This formula adjusts for overestimation by including reported counts in the denominator [5,38].

Resulting interoceptive accuracy scores were averaged across the six trials, yielding a mean score for each participant. Accuracy scores can range from −1 to 1, with higher scores indicating greater accuracy. Interoceptive accuracy data from 11 girls were missing owing to technical issues. The mean interoceptive accuracy score was −0.28 (s.d. = 0.58).

### (d) Pubertal developmental scale

The PDS (girls version) [35] was used at the end of the study to assess pubertal stage. The parent- and self-reported PDS shows strong internal consistency, test–retest reliability and good correspondence with Tanner staging [39]. It includes five items—growth spurt, body hair, breast development, skin changes and menarche—each rated on a 4-point scale (1 = not yet started, 2 = barely started, 3 = underway, 4 = complete), except menarche (1 = no, 4 = yes). An average score of all five items is computed, creating a mean score ranging from 1 to 4, with the highest score of 4 indicating that the individual is post-puberty. Data were missing for 38 girls owing to lack of parental response. In the present sample, the average PDS score was 2.53 (s.d. = 0.78), ranging from 1 to 3.6, indicating broad variation from earlier to more advanced pubertal stages.

### (e) Heart rate

Heart rate was calculated as the average across the six trials of the HCT. All values fell within a plausible range (50–120 beats per minute (bpm)). Data from 12 participants were missing owing to technical issues. The mean heart rate was 84.81 bpm (s.d. = 10.63).

### (f) Body mass index

BMI was estimated using the Pedi Quick Weight App, based on the participant's age (years and months), gender, height and perceived body composition. These details, including the girl's height, were collected at the end of each testing session. We opted for this estimation approach instead of directly weighing participants, to avoid potential harm or discomfort during this sensitive transitional developmental period. Values below 10 or above 60 were considered implausible and coded as missing. BMI data were missing for 78 participants owing to human error. The average BMI was 18.16 (s.d. = 3.42).

### (g) Statistical analyses

All analyses were carried out in Stata 15. Interoceptive accuracy and pubertal stage were treated as primary predictors, with pubertal stage also specified as a moderator via the interaction term; BIAS and BIQ were analysed as separate outcome variables in parallel models.

First, to examine whether interoceptive accuracy predicted behavioural body dissatisfaction (BIAS scores) and whether this was moderated by pubertal stage, we ran a multiple regression with interoceptive accuracy and pubertal stage as mean-centred continuous predictors, including their interaction. Model diagnostics included inspection of residual-versus-fitted plots, quantile–quantile plots of residuals, the Breusch-Pagan/Cook-Weisberg test for heteroskedasticity, and influence diagnostics using Cook's distance and leverage values. Because some study variables departed from normality, and to reduce reliance on normal-theory inference, we used bootstrap resampling with 1000 replications (seed = 333) for the primary complete-case models. The results reported in the main text for the primary complete-case models are based on ordinary least squares coefficient estimates with bootstrap-derived standard errors, confidence intervals (CIs) and associated *p*-values. Because the Breusch-Pagan/Cook-Weisberg test indicated heteroskedasticity in the BIAS model, we conducted an additional sensitivity analysis using heteroskedasticity-robust standard errors via the vce(robust) option in Stata. This yielded the same overall pattern of results. Influence diagnostics did not suggest that the results were driven by any single highly influential observation. Significant interactions were followed up by plotting values at low (−1 s.d.) and high (+1 s.d.) scores and examining the differences [40]. These analyses were conducted on a sample of 129 participants, excluding those with missing data on variables included in the model. Given that missingness in this primary complete-case model was limited relative to the covariate-adjusted analyses (see below), we opted for listwise deletion to maintain analytical simplicity.

To account for key potential confounding variables, we then repeated the analyses, including heart rate and BMI as covariates. Pubertal stage was the focal moderator in the primary models, whereas BMI and heart rate were included only as secondary covariates in sensitivity analyses. Owing to high BMI missingness (43.1%), which raised concerns about bias, we used multiple imputation by chained equations (MICE) with predictive mean matching (*k*nn = 5), generating 20 imputations. MICE is widely recommended for handling missing data under a missing at random (MAR) assumption and can perform adequately even with relatively high missingness, although estimates become increasingly sensitive to model specification as missingness increases [41,42]. In our data, BMI missingness was related to observed variables such as age, pubertal stage, interoceptive accuracy and body dissatisfaction. Therefore, BMI and heart rate were imputed using these observed variables, supporting the plausibility of a MAR mechanism conditional on observed data. These covariate-adjusted models were treated as sensitivity analyses. Missing BMI and heart rate values were imputed, so cases missing on other variables included in a given model were still excluded (final analytic sample sizes for each model are reported in the supplementary material, table S9). All imputed values were within expected bounds and distributions were stable. Monte Carlo errors were below 10% of their standard errors, indicating low imputation uncertainty [43].

Second, to examine whether interoceptive accuracy predicted self-reported body satisfaction and whether this was moderated by pubertal stage, we repeated the same models using the self-reported BIQ as the outcome. Analyses without covariates used the same bootstrap approach. For the BIQ model, diagnostic checks did not indicate major violations, and the Breusch-Pagan/Cook-Weisberg test did not provide evidence of substantial heteroskedasticity. Influence diagnostics likewise did not suggest that results were driven by any single highly influential observation. These analyses were conducted on a sample of 133 participants, excluding those with missing data on variables included in the model. When adding heart rate and BMI as covariates, MICE was again used for BMI and heart rate (20 imputations, *k*nn = 5), incorporating observed variables associated with BMI missingness, with the same quality checks confirming validity. Final analytic sample sizes for these models are reported in the supplementary material, table S9.

We conducted additional robustness analyses, including age as a covariate, and inspected multicollinearity diagnostics. Full results are reported in the supplementary material.

## 4. Results

### (a) Descriptive statistics

The mean BIAS score was 3.81 (s.d. = 4.23), and the mean BIQ score was 0.99 (s.d. = 1.38). The mean score for puberty stage was 2.51 (s.d. = 0.77; range 1 to 3.6; figure 1B), indicating broad variation across the sample. Zero-order correlations were computed using Pearson's *r* on complete-case data across the variables included in the correlation matrix (*n* = 125) shown in figure 1A. Visual inspection of scatterplots and checks for extreme values did not indicate violations of linearity or undue influence of outliers. BIAS and BIQ scores were positively correlated, *r* = 0.53, *p* < 0.001. Interoceptive accuracy was negatively correlated with both BIAS scores, *r* = −0.24, *p* = 0.006 and BIQ scores, *r* = −0.23, *p* = 0.010. Pubertal stage and age were positively correlated, *r* = 0.66, *p* < 0.001, consistent with previous studies [44]. Interoceptive accuracy was negatively correlated with both pubertal stage, *r* = −0.19, *p* = 0.031 and age, *r* = −0.26, *p* = 0.004. These zero-order correlations are reported for descriptive purposes only; our primary hypotheses were tested in the regression models described below.

**Figure 1.**
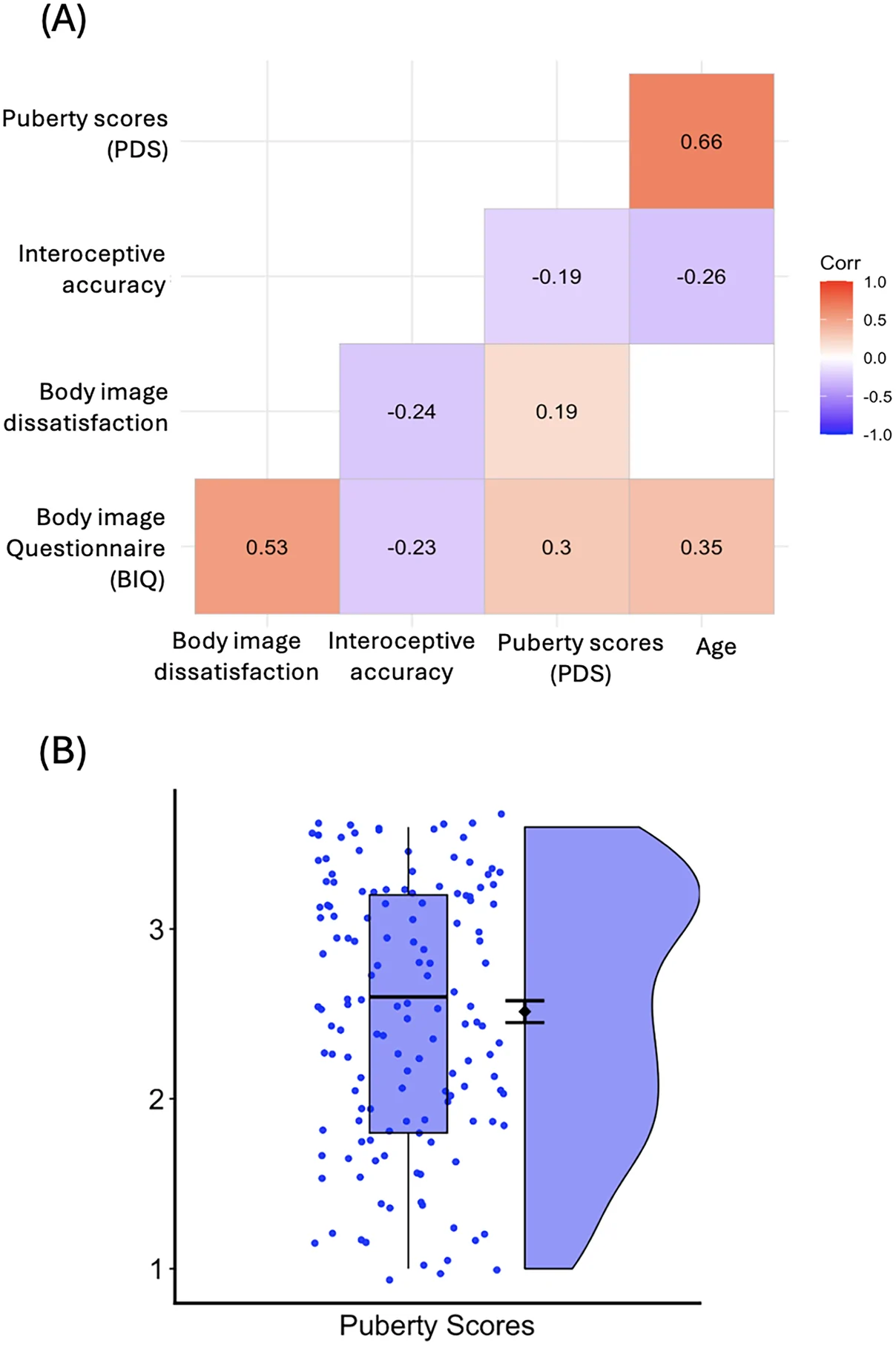
(A) Zero-order correlation matrix of the main variables. Blank squares indicate non-significant correlations; coloured squares represent significant correlations, with red hues indicating more positive coefficients and purple hues indicating more negative ones. Correlation values are displayed within the squares. (B) Distribution of PDS scores shown via raincloud plots, combining raw jittered data, boxplots for central tendency and density distributions. Error bars represent the s.e.m.

### (b) Main analyses

#### (i) Is interoceptive accuracy associated with body satisfaction across pubertal stages?

We first answer this question by looking at the relationship between interoceptive accuracy and body dissatisfaction as measured behaviourally (BIAS). Interoceptive accuracy significantly predicted lower body dissatisfaction, *b* = −1.57, s.e. = 0.63, *p* = 0.013, 95% CI (−2.81, −0.34), indicating that higher interoceptive accuracy was associated with lower dissatisfaction, independent of pubertal stage. Pubertal stage also emerged as a significant positive predictor, *b* = 0.82, s.e. = 0.38, *p* = 0.031, 95% CI (0.07, 1.57), as girls who were further advanced in puberty had greater body dissatisfaction. As hypothesized, we found a significant interoceptive accuracy by pubertal stage interaction, *b =* −1.57, s.e. = 0.67, *p* = 0.018, 95% CI (−2.88, −0.26), supporting the hypothesis that the relationship between interoceptive accuracy and body dissatisfaction varies across stages of puberty.

To follow-up the significant interaction, we examined body-image dissatisfaction at low (−1 s.d.) and high (+1 s.d.) levels of interoceptive accuracy and pubertal stage. These showed that higher body-image dissatisfaction is observed for lower, relative to higher, levels of interoceptive accuracy during more advanced puberty stages, *b =* 3.25, s.e. = 1.21, *p* = 0.007. By contrast, there was no difference in body-image dissatisfaction between those with high and low interoceptive accuracy during early puberty stages, *b =* 0.42, s.e. = 0.59, *p* = 0.472; figure 2A. Thus, it seems that the protective role of higher interoceptive accuracy occurs only at later stages of puberty. Indeed, as shown in figure 2B, lower interoceptive accuracy at later puberty stages was associated with greater body-image dissatisfaction.

**Figure 2.**
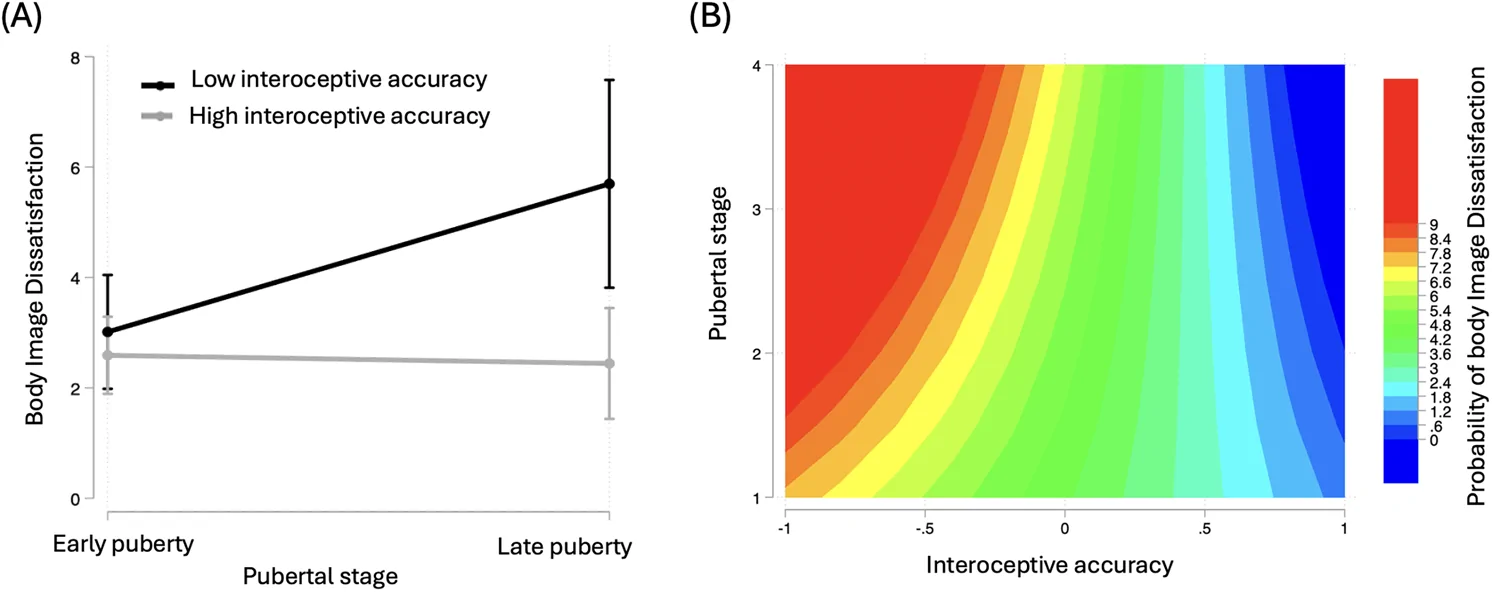
(A) Interoceptive accuracy by pubertal stage on the body-image dissatisfaction scores from the BIAS task. Values are plotted for BIAS scores at low (−1 s.d.) and high (+1 s.d.) interoceptive accuracy and at early (−1 s.d.) and late (+1 s.d.) pubertal stage. Interoceptive accuracy had no significant effect at early puberty, *b* = 0.42, s.e. = 0.59, *p* = 0.472, but at later stages, girls with higher interoceptive accuracy showed significantly lower body dissatisfaction compared with those with lower accuracy, *b* = 3.25, s.e. = 1.21, *p* = 0.007. (B) Heatmap showing predicted body-image dissatisfaction as a function of interoceptive accuracy and pubertal stage. Red indicates higher predicted body-image dissatisfaction, and blue, lower predicted dissatisfaction. The effect of interoceptive accuracy is most pronounced at later stages of puberty, where lower interoceptive accuracy is associated with higher dissatisfaction.

When controlling for BMI and heart rate as covariates and using multiply imputed data (*m* = 20), we observed the same pattern of results with respect to the interaction between puberty and interoceptive accuracy, *b =* −1.53, s.e. = 0.70, *p* = 0.032, as well as a main effect of interoceptive accuracy *b =* −1.53, s.e. = 0.59, *p* = 0.011. However, no significant main effects were observed for puberty stage (*p* = 0.090), BMI (*p* = 0.490), or heart rate (*p* = 0.236). See the supplementary material, table S1 for results and Monte Carlo error diagnostics. In additional robustness analyses, including age as a covariate, the interoceptive accuracy by pubertal stage interaction remained significant, whereas age was not a significant predictor, and multicollinearity diagnostics were low (see the supplementary material, tables S3, S4 and S7).

#### (ii) Is interoceptive accuracy associated with self-reported body satisfaction across pubertal stages?

We answer this question by looking at the relationship between interoceptive accuracy and body satisfaction as measured by the self-report. Puberty stage significantly predicted higher BIQ scores, *b =* 0.43, s.e. = 0.13, *p* = 0.001, 95% CI (0.18, 0.69), indicating that girls who were further advanced in puberty had greater body-image dissatisfaction. Interoceptive accuracy also significantly predicted lower BIQ scores, *b =* −0.42, s.e. = 0.20, *p* = 0.041, 95% CI (−0.82, −0.02), suggesting that girls with higher interoceptive accuracy reported less body dissatisfaction. However, the interaction between interoceptive accuracy and puberty stage was non-significant, *b =* −0.29, s.e. = 0.25, *p* = 0.248, 95% CI (−0.77, 0.20). Although the direction of effects mirrors those found with the behavioural BIAS measure (see figure 3), the lack of interaction suggests weaker evidence that interoceptive accuracy moderates body dissatisfaction across puberty stages at the self-report level. Indeed, a Bayes factor analysis (BF_10_ = 0.04) provided strong support for the null hypothesis.

**Figure 3.**
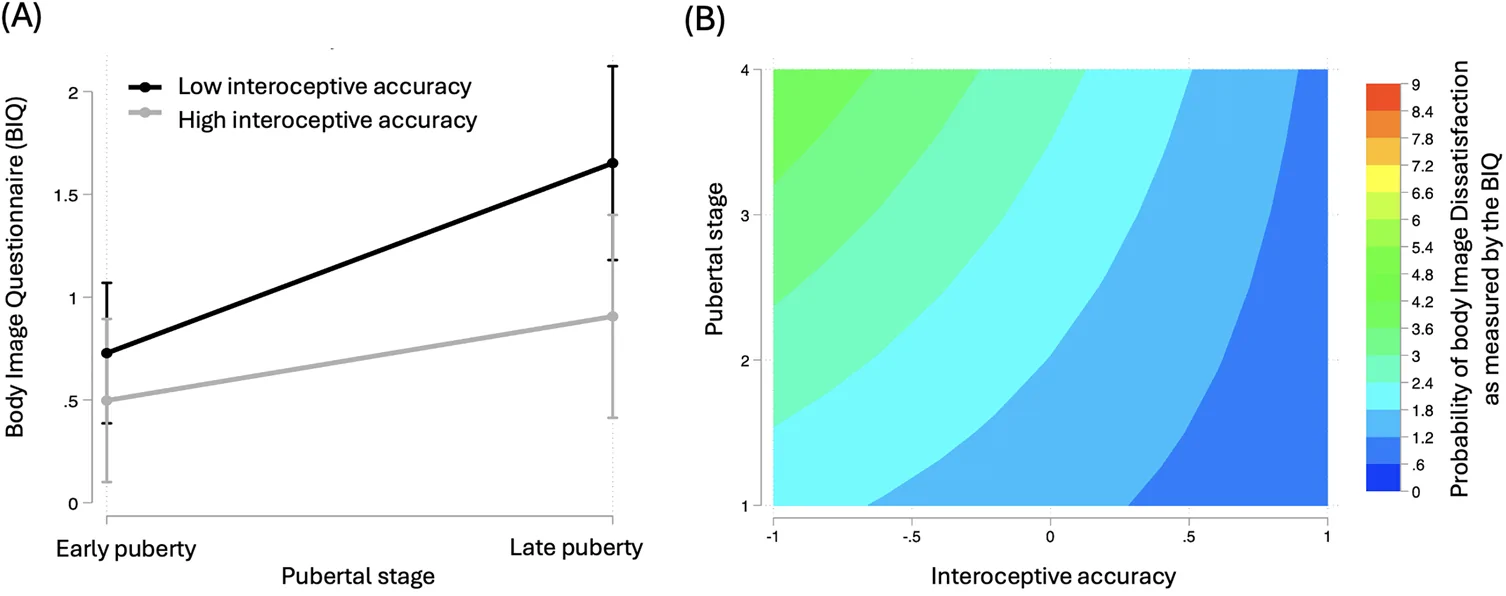
Interoceptive accuracy by pubertal stage on the BIQ. We observe a similar pattern to that in figure 2. However, this interaction was non-significant, *b* = −0.29, s.e. = 0.25, *p* = 0.248.

When controlling for BMI and heart rate as covariates and using multiply imputed data (*m* = 20), we found the same pattern of results. Interoceptive accuracy remained a significant negative predictor of body dissatisfaction, *b =* −0.40, s.e. = 0.19, *p* = 0.041, puberty stage was again a significant positive predictor, *b =* 0.40, s.e. = 0.16, *p* = 0.015, 95% CI (0.08, 0.73), and the interaction between interoceptive accuracy and pubertal stage was not significant, *b =* −0.30, s.e. = 0.23, *p* = 0.205, suggesting no moderation effect. No significant main effects were observed for BMI (*p* = 0.309) or heart rate (*p* = 0.554). Full results and Monte Carlo error diagnostics are provided in the supplementary material, table S2. Additional robustness analyses, including age as a covariate, showed the same overall pattern: the interoceptive accuracy by pubertal stage interaction remained non-significant, whereas age showed a small positive association with BIQ scores. Multicollinearity diagnostics were low (see the supplementary material, tables S5–S7).

## 5. Discussion

We investigated how interoceptive accuracy and pubertal developmental stage were associated with two measures of body-image dissatisfaction. Across both behavioural (BIAS task) and self-reported (BIQ) measures, lower levels of interoceptive accuracy were consistently associated with greater body-image dissatisfaction. This overall finding aligns with previous studies conducted in older populations [7,9–11,14,45], suggesting that greater interoceptive accuracy generally plays a protective role against negative body image. However, our study provides novel evidence by demonstrating an important moderation effect: the protective influence of interoceptive accuracy on body-image dissatisfaction depended on the pubertal developmental stage, at least at the behavioural level. Specifically, this beneficial effect of interoception was particularly pronounced in girls at more advanced stages of puberty. We discuss these findings below.

Overall, we found that higher levels of interoceptive accuracy predicted less body-image dissatisfaction, regardless of puberty stage, for both behavioural (BIAS) and self-report (BIQ) measures. These findings are broadly consistent with previous research, indicating that interoceptive accuracy can help mitigate negative body image across different stages of life [7,9]. Importantly, however, our findings uniquely extend this literature to a younger, female, pubertal population, suggesting that the protective role of interoceptive accuracy may already be evident in early adolescence and may be particularly relevant among girls at more advanced pubertal stages. This developmental period is characterized by rapid and significant bodily changes and increasing susceptibility to body dissatisfaction [26]. Thus, interoceptive accuracy, and perhaps interoceptive awareness more broadly, may provide an important internal mental representation of the body and the self that helps adolescents maintain body satisfaction amidst these physical and psychosocial changes.

In line with recent proposals [7,16,19], one possible explanation is that girls undergoing puberty may particularly benefit from relying more on their relatively accurate interoceptive signals about how they feel, rather than on exteroceptive cues concerning how they look. Owing to rapid and often unpredictable changes in body shape during puberty, exteroceptive cues (e.g. external appearance, comparisons with peers and societal body ideals) can become unreliable or unstable. Furthermore, the broader psychosocial context of peer pressure and the internalization of unrealistic body ideals may further exacerbate the tendency to focus on one's appearance and objectification. In this context, individuals with a strong, accurate, internal bodily perception (good interoception) may maintain a more stable body image and be less influenced by such psychosocial pressures. Conversely, individuals with poorer interoceptive accuracy may become more vulnerable to negative influences. Therefore, interoception may serve as a crucial protective factor during the physiological and social challenges of puberty. Supporting this view, a recent study by Riboli *et al.* [13] showed that a self-report measure of interoceptive accuracy mediates the relationship between body image and mental health in adolescents, highlighting the importance of bodily awareness for psychological resilience during this developmental period.

Critically, our findings also suggest this protective role of interoceptive accuracy is particularly relevant and important among girls at later stages of puberty, when vulnerability to negative body image increases [29]. We propose two explanations for the absence of such an effect in early puberty. First, it is possible that interoceptive accuracy is not fully developed in early puberty and thus does not affect body dissatisfaction so prominently at this stage. Although interoception during this developmental period has not been studied, it has been suggested that interoception is poorer in early stages compared with later stages of puberty and adolescence. In fact, puberty may serve as a sensitive period for interoceptive learning. Specifically, Murphy *et al.* [46] argue that, if associative learning is crucial in infancy for the contextualization of interoceptive cues [47], then, during puberty, a time of significant bodily changes, associative learning may be essential for adapting to new, unexpected visceral sensations. However, we consider this unlikely, as we would expect to see an increase in interoceptive accuracy across pubertal stages if it were a period conducive to interoceptive learning. Instead, our data show that higher pubertal stage correlates with lower interoceptive accuracy. Alternatively, it is possible that at later pubertal stages adolescents become more vulnerable to body-image concerns [29]. Our data support this interpretation by showing that later pubertal stages are associated with increased body dissatisfaction. Therefore, we propose that the benefits of interoceptive accuracy on body image become more pronounced as puberty progresses, as it is then that the risk of developing body dissatisfaction emerges.

Even though this effect was not observed at a self-report level, interoceptive accuracy was still associated with lower body dissatisfaction in the BIQ, replicating previous findings in adolescent populations using the same questionnaire [9]. Although both measures are self-referential, the BIAS may capture the perception of the body (ideal) more directly, given that this is a behavioural task that allows us to quantify body-image distortions and body dissatisfaction, whereas the BIQ indexes explicit self-report and evaluation of body appearance. One possibility is that pubertal moderation operates more strongly at a perceptual level, as captured by the behavioural BIAS measure, than at the level of explicit self-evaluation indexed by self-report. In other words, interoceptive accuracy may shape body representation before it is fully reflected in conscious appraisal. At the same time, because a similar descriptive pattern emerged for the BIQ, limited power may also have contributed to the null interaction. Future research should test these possibilities in larger samples. Given growing interest in sex differences in interoception [48] and the prevalence of body-image concerns in boys [49], including boys could also offer a more comprehensive view of how interoceptive accuracy interacts with sex and pubertal development in shaping body-image dissatisfaction.

Despite these insights, our findings should be considered in light of their limitations and directions for future research. First, although the study was initially designed to be longitudinal, COVID-19 restrictions forced us to adopt a cross-sectional design, precluding any direct test of causality. Consequently, cohort effects, secular trends and confounding variables related to individual differences may have influenced the observed associations and may limit their generalizability. Future longitudinal studies that control for individual differences and better capture changes over time are needed to establish temporal precedence and causality. In addition, because data collection spanned periods before and after the onset of COVID-19 restrictions, broader pandemic-related contextual factors may also have influenced body image and interoceptive processes. This should be taken into account when interpreting the findings. For example, population-based studies have shown an increased incidence of eating disorders diagnosis among teenage girls in the UK during the first 2 years of the COVID-19 pandemic [50]. Against this backdrop, our findings suggest that interoception may still play a relevant role in body image during adolescence even when external societal factors may exacerbate body-image concerns.

Second, although the present study examined behavioural associations only and did not test neural mechanisms directly, some effects observed may reflect underlying brain changes during puberty. Incorporating neuroimaging could help clarify how the developing brain shapes the relationship between interoception and body image. Future studies could track changes from pre- to post-puberty in how the self perceives the body, alongside the maturation of interoceptive and social cognition networks. The anterior insula, central to interoception, undergoes significant reorganization during puberty. Longitudinal magnetic resonance imaging (MRI) shows continued cortical grey matter thinning and increases in white matter volume [51], while resting-state studies report age-related strengthening of salience network connectivity centred on the right anterior insula [52]. Task-based functional MRI further shows that pubertal stage modulates insular activity during interoceptive tasks [27]. In adults, insular connectivity supports integration of interoceptive and exteroceptive signals to form bodily self-representations [53], and anterior insula volume predicts interoceptive accuracy [54]. Building on this, future research combining behavioural and neuroimaging approaches should test whether structural and functional maturation of the anterior insula mediates puberty-related differences in interoceptive accuracy that may, in turn, protect against body-image dissatisfaction.

Third, considering the established relationship between BMI and aspects of interoception [55] as well as body image [56], we controlled for BMI in our analyses and found the same pattern of results. However, the significant amount of missing BMI data represents a notable limitation in this study. This challenge is frequently encountered in research involving adolescent samples [57] and future research is needed to address this limitation.

Finally, the HCT should be interpreted in light of the task's recognized limitations. Although it is widely used as a behavioural index of cardiac interoceptive performance, it has been criticized for potential confounds, including cardiac knowledge, time estimation and task-related strategies [58]. These concerns may be particularly relevant in adolescence, when broader cognitive factors, such as attention, are still developing. In the present study, we attempted to minimize reliance on time estimation by instructing participants to count only heartbeats they could genuinely feel rather than estimate [37], although non-interoceptive influences may still have contributed to performance. Importantly, HCT performance has also been linked to theoretically relevant outcomes, including body image and eating-disordered symptomatology [7,8,24]. Recent evidence also suggests that HCT performance shows some convergence with other cardiac interoceptive measures and is not fully explained by time estimation alone [59]. Thus, although the HCT has recognized limitations, it remains a useful behavioural proxy of cardiac interoceptive performance. Future work would benefit from combining it with complementary behavioural and self-report measures across puberty.

## 6. Conclusion

In summary, our findings suggest that interoceptive accuracy influences body-image dissatisfaction depending on pubertal stage, at least at a behavioural level. Using the BIAS, we found that in late, but not early puberty, lower interoceptive accuracy predicted greater body dissatisfaction. We propose that interoceptive accuracy may facilitate how well girls navigate body-image concerns throughout the many physiological changes associated with puberty.

## Supplementary Material



## Data Availability

Data, code and materials are available at the Open Science Framework: https://osf.io/fwp8y/. Supplementary material is available online [60].

## Theme

One contribution of 17 to a discussion meeting issue ‘From being to identity: analysis and synthesis of the self ’.
