# Supplementary material for: Tuning in: how interoception shapes body image across early adolescence
Source: Philos Trans R Soc Lond B Biol Sci. 2026 Nov 10;381(1962):20250159. doi: 10.1098/rstb.2025.0159 (PMC13750425; doi:10.1098/rstb.2025.0159)
Supplement: Supplementary file 1 [file rstb.2025.0159.zip › rstb.2025.0159/rstb20250159_si_001.docx]

**Supplementary Materials**

*Tuning In: How Interoception Shapes Body Image Across Early Adolescence*

Mariana von Mohr, Rosie Donaghy, Vivien Ainley, Imogen Moore, Abigail Agyemang, Manos Tsakiris

## Table S1. Regression results for Body Image Dissatisfaction (BIAS) scores using multiple imputation with Monte Carlo error estimates.

Regression results from the multiply imputed dataset (m = 20) including BMI and heart rate as covariates. Monte Carlo error estimates are reported for transparency. Number of observations = 126.

| **Predictor** | **b** | **SE** | **p** | **MC Error** | **MC SE** | **95% CI** |
| --- | --- | --- | --- | --- | --- | --- |
| Interoceptive accuracy | -1.53 | 0.59 | .011 | 0.0092 | 0.0009 | -2.71 to -0.36 |
| Pubertal development | 0.84 | 0.49 | .090 | 0.0173 | 0.0036 | -0.13 to 1.80 |
| Accuracy x Puberty | -1.53 | 0.70 | .032 | 0.0098 | 0.0012 | -2.92 to -0.13 |
| BMI | 0.08 | 0.11 | .490 | 0.0106 | 0.0048 | -0.15 to 0.31 |
| Heart rate | 0.04 | 0.03 | .236 | 0.0008 | 0.0001 | -0.02 to 0.10 |
| Intercept | -1.13 | 2.95 | .703 | 0.1376 | 0.0280 | -6.96 to 4.71 |

## Table S2. Regression results for Body Image Questionnaire (BIQ) scores using multiple imputation with Monte Carlo error estimates.

Regression results from the multiply imputed dataset (m = 20) including BMI and heart rate as covariates. Number of observations = 127.

| **Predictor** | **b** | **SE** | **p** | **MC Error** | **MC SE** | **95% CI** |
| --- | --- | --- | --- | --- | --- | --- |
| Interoceptive accuracy | -0.40 | 0.19 | .041 | 0.0055 | 0.0007 | -0.78 to -0.02 |
| Pubertal development | 0.40 | 0.16 | .015 | 0.0104 | 0.0024 | 0.08 to 0.73 |
| Accuracy x Puberty | -0.30 | 0.23 | .205 | 0.0097 | 0.0017 | -0.75 to 0.16 |
| BMI | 0.05 | 0.04 | .309 | 0.0066 | 0.0038 | -0.04 to 0.14 |
| Heart rate | 0.01 | 0.01 | .554 | 0.0005 | 0.0001 | -0.01 to 0.03 |
| Intercept | -0.45 | 1.01 | .661 | 0.0825 | 0.0218 | -2.46 to 1.57 |

## Table S3. Age-adjusted complete-case regression results for BIAS scores.

Bootstrap-estimated regression model including age as a covariate (1,000 replications, seed = 333). Number of observations = 126.

| **Predictor** | **b** | **SE** | **p** | **95% CI** |
| --- | --- | --- | --- | --- |
| Interoceptive accuracy | -1.60 | 0.62 | .011 | -2.82 to -0.37 |
| Pubertal development | 0.73 | 0.53 | .170 | -0.31 to 1.78 |
| Accuracy x Puberty | -1.56 | 0.69 | .024 | -2.92 to -0.21 |
| Age | 0.11 | 0.42 | .792 | -0.71 to 0.93 |
| Intercept | 3.43 | 0.31 | <.001 | 2.83 to 4.04 |

## Table S4. Age-adjusted multiple-imputation regression results for BIAS scores.

Sensitivity model including age, BMI, and heart rate as covariates in the multiply imputed dataset (m = 20). Number of observations = 125.

| **Predictor** | **b** | **SE** | **p** | **MC Error** | **MC SE** | **95% CI** |
| --- | --- | --- | --- | --- | --- | --- |
| Interoceptive accuracy | -1.57 | 0.61 | .011 | 0.0120 | 0.0016 | -2.78 to -0.36 |
| Pubertal development | 0.70 | 0.60 | .247 | 0.0142 | 0.0021 | -0.49 to 1.89 |
| Accuracy x Puberty | -1.52 | 0.71 | .034 | 0.0112 | 0.0013 | -2.93 to -0.12 |
| Age | 0.11 | 0.37 | .760 | 0.0072 | 0.0012 | -0.62 to 0.85 |
| BMI | 0.09 | 0.12 | .446 | 0.0114 | 0.0052 | -0.14 to 0.33 |
| Heart rate | 0.04 | 0.03 | .265 | 0.0009 | 0.0001 | -0.03 to 0.10 |
| Intercept | -1.26 | 2.97 | .673 | 0.1417 | 0.0289 | -7.13 to 4.62 |

## Table S5. Age-adjusted complete-case regression results for BIQ scores.

Bootstrap-estimated regression model including age as a covariate (1,000 replications, seed = 333). Number of observations = 131.

| **Predictor** | **b** | **SE** | **p** | **95% CI** |
| --- | --- | --- | --- | --- |
| Interoceptive accuracy | -0.37 | 0.20 | .067 | -0.77 to 0.03 |
| Pubertal development | 0.19 | 0.19 | .311 | -0.18 to 0.55 |
| Accuracy x Puberty | -0.30 | 0.24 | .218 | -0.77 to 0.18 |
| Age | 0.24 | 0.12 | .055 | -0.01 to 0.48 |
| Intercept | 0.93 | 0.10 | <.001 | 0.73 to 1.14 |

## Table S6. Age-adjusted multiple-imputation regression results for BIQ scores.

Sensitivity model including age, BMI, and heart rate as covariates in the multiply imputed dataset (m = 20). Number of observations = 126.

| **Predictor** | **b** | **SE** | **p** | **MC Error** | **MC SE** | **95% CI** |
| --- | --- | --- | --- | --- | --- | --- |
| Interoceptive accuracy | -0.35 | 0.19 | .070 | 0.0047 | 0.0009 | -0.74 to 0.03 |
| Pubertal development | 0.16 | 0.19 | .396 | 0.0082 | 0.0010 | -0.22 to 0.54 |
| Accuracy x Puberty | -0.31 | 0.23 | .172 | 0.0099 | 0.0018 | -0.77 to 0.14 |
| Age | 0.24 | 0.12 | .039 | 0.0034 | 0.0004 | 0.01 to 0.47 |
| BMI | 0.05 | 0.05 | .324 | 0.0066 | 0.0037 | -0.05 to 0.14 |
| Heart rate | 0.01 | 0.01 | .459 | 0.0006 | 0.0001 | -0.01 to 0.03 |
| Intercept | -0.57 | 0.99 | .565 | 0.0794 | 0.0187 | -2.55 to 1.40 |

## Table S7. Multicollinearity diagnostics for the age-adjusted complete-case models.

Variance inflation factors (VIFs) were low in both models, indicating no concerning multicollinearity between age, pubertal development, and interoceptive accuracy.

| **Variable** | **BIAS VIF** | **BIQ VIF** |
| --- | --- | --- |
| Interoceptive accuracy | 1.10 | 1.10 |
| Pubertal development | 1.78 | 1.76 |
| Accuracy x Puberty | 1.04 | 1.03 |
| Age | 1.85 | 1.82 |
| Mean VIF | 1.44 | 1.43 |

## Table S8. Missing-data summary and analytic sample sizes by model.

Missingness overlapped across variables, so variable-level missing counts do not sum directly to the number of excluded cases per model. Multiple imputation was applied only to BMI and heart rate; therefore, cases missing on other variables included in a given model were still excluded from that analysis. Final analytic sample sizes for each model are reported in Table S9.

| **Measure / model** | **Observed N** | **Missing N** |
| --- | --- | --- |
| Original sample | 181 | 0 |
| BIAS outcome | 169 | 12 |
| BIQ outcome | 177 | 4 |
| Interoceptive accuracy | 170 | 11 |
| Pubertal development | 143 | 38 |
| Age | 177 | 4 |
| BMI | 103 | 78 |
| Heart rate | 169 | 12 |
| BIAS primary model (manuscript) | 129 | 52 |
| BIAS MI model with BMI + HR | 126 | 55 |
| BIAS age-adjusted complete-case model | 126 | 55 |
| BIAS age-adjusted MI model | 125 | 56 |
| BIQ primary model (manuscript) | 133 | 48 |
| BIQ MI model with BMI + HR | 127 | 54 |
| BIQ age-adjusted complete-case model | 131 | 50 |
| BIQ age-adjusted MI model | 126 | 55 |

## Note. This table summarises observed and missing values before multiple imputation. The starting analytic dataset contained 181 participants after removal of 11 fully empty records from the original file. Missingness overlapped across variables, so variable-level missing counts do not sum directly to the number of excluded cases per model.

**Table S9. Analytic sample sizes, inclusion criteria, and missing-data handling by model**

| **Analysis** | **Outcome** | **Predictors / covariates** | **Missing-data approach** | **Final N** | **Inclusion / exclusion rule** |
| --- | --- | --- | --- | --- | --- |
| Primary model | BIAS | IAcc, PDS, IAcc × PDS | Complete-case | 129 | Excluded if missing BIAS, IAcc, or PDS |
| Primary model | BIQ | IAcc, PDS, IAcc × PDS | Complete-case | 133 | Excluded if missing BIQ, IAcc, or PDS |
| Covariate-adjusted sensitivity model | BIAS | IAcc, PDS, IAcc × PDS, BMI, HR | MI for BMI and HR only | 126 | BMI and HR imputed; cases missing BIAS, IAcc, or PDS still excluded |
| Covariate-adjusted sensitivity model | BIQ | IAcc, PDS, IAcc × PDS, BMI, HR | MI for BMI and HR only | 127 | BMI and HR imputed; cases missing BIQ, IAcc, or PDS still excluded |
| Age-adjusted robustness model | BIAS | IAcc, PDS, IAcc × PDS, Age | Complete-case | 126 | Excluded if missing BIAS, IAcc, PDS, or Age |
| Age-adjusted robustness model | BIQ | IAcc, PDS, IAcc × PDS, Age | Complete-case | 131 | Excluded if missing BIQ, IAcc, PDS, or Age |
| Age-adjusted MI model | BIAS | IAcc, PDS, IAcc × PDS, Age, BMI, HR | MI for BMI and HR only | 125 | BMI and HR imputed; cases missing BIAS, IAcc, PDS, or Age still excluded |
| Age-adjusted MI model | BIQ | IAcc, PDS, IAcc × PDS, Age, BMI, HR | MI for BMI and HR only | 126 | BMI and HR imputed; cases missing BIQ, IAcc, PDS, or Age still excluded |

## ****Note.**** Final N refers to the estimation sample for each fitted model. Multiple imputation was applied only to BMI and heart rate. Outcome variables and primary predictors were not imputed, so cases missing on non-imputed variables were still excluded from the relevant model. Variable-level missingness in the pre-imputation analytic dataset is reported in Table S8.
